# Ethnic Disparities in Social Capital and Health among Jewish and Arab Participants in the Israeli Mamanet Cachibol League

**DOI:** 10.3390/ijerph18010295

**Published:** 2021-01-03

**Authors:** Yuval Paldi, Daniel S. Moran, Orna Baron-Epel, Shiran Bord, Riki Tesler

**Affiliations:** 1 Department of Health Systems Management, Faculty of Health Science, Ariel University, Ariel 40700, Israel; danielm@ariel.ac.il (D.S.M.); riki.tesler@gmail.com (R.T.); 2 School of Public Health, Faculty of Social Welfare and Health Sciences, University of Haifa, Haifa 31905, Israel; ornaepel@research.haifa.ac.il; 3 Department of Health Systems Management, The Max Stern Yezreel Valley College, Emek Yezreel 1930600, Israel; shiranb@yvc.ac.il

**Keywords:** Arab and Jewish women, amateur team sports, well-being, self-reported health, social capital

## Abstract

The Israeli Mamanet Cachibol League (MCL) serves as a community model that incorporates physical activity and amateur team sports among women. Team sports have been shown to bridge gaps and build positive relationships between communities. There is a paucity of data regarding the advantages of team sports to promote the health and well-being of women from different ethnic backgrounds. The purpose of this study was to examine the association of participation in MCL with social capital, health, and well-being across two ethnic groups: Jewish and Arab women. A cross-sectional survey was conducted among women aged 25–64: 102 Jewish and 96 Arab MCL participants, and 102 Jewish and 81 Arab non-MCL participants. Data regarding social capital (trust, social support and social involvement) and well-being (self-reported health and psychosomatic and depressive symptoms) were analyzed using two-way analyses of covariance and multiple regression models with sequential entry of the variables. MCL participants from both ethnic groups reported higher social capital (*p* < 0.001), better self-reported health (*p* < 0.001), and lower psychosomatic symptoms (*p* < 0.001) compared to non-participants. Jewish MCL participants reported lower depressive symptoms (*p* < 0.001) than non-participants, however no difference was found between Arab MCL participants and non-participants (*p* < 0.160). Amateur team sports such as MCL are related with higher levels of well-being and social capital. Future research should focus on longitudinal studies that examine the change in social capital and well-being over time.

## 1. Introduction

In recent years, a considerable body of literature on social capital has emerged linking social capital to a number of positive outcomes, including health indicators and well-being [1,2]. Social capital includes social networks, social involvement, norms, and trust, all of which enable coordination and cooperation among groups or communities in return for mutual benefits [3]. High social capital is a result of tight social networks, characterized by trust and reciprocity between members of the group or community [4,5]. Putnam [4] suggested five indicators of social capital: (1) social involvement, (2) political involvement (participating and attitudes), (3) volunteering and reciprocity, (4) trust, and (5) social support. In the current study, we adopted Putnam’s definition of social capital: “features of social organization such as networks, norms, and social trust that facilitate coordination and cooperation for mutual benefit” [4], as it positions participation in structured networks, such as sports clubs, as enhancing a productive, supportive, trusting, and effective society for the benefit of the wider population.

Well-being describes a wide range of ways in which people experience and express their lives in either a positive or a non-negative manner. There are those who equate well-being with bliss and joy, others view well-being as a state of sustained contentment, and for others it is simply a state of good physical and mental health. All of these views are correct but none of them stands alone, meaning that well-being cannot be fully represented by any one measure [6].

There are two major conceptual approaches to well-being [7]. In the first approach, one emphasizes the individual’s assessment of his or her own life, both emotionally and cognitively. This approach has been referred as hedonic well-being (HWB) or subjective well-being as it emphasizes internal rather than external assessment [8].

The second approach consists of several concepts, which have been referred to as eudaimonic well-being (EWB). This approach assumes there are a number of needs or qualities essential for one’s psychological growth and development. Fulfillment of these needs enables individuals to reach their full potential [7]. Another EWB approach emphasizes the importance of living up to one’s potential in the sense of pursuing goals and activities in accordance with one’s values and identity [9].

Among women, social capital has been found to be associated with well-being: higher levels of happiness, life satisfaction [10,11], quality of life and reduced risk for anxiety and depression [12,13]. In addition, social capital was found to be associated with engaging in physical activity (PA) and healthy behaviors among women of various ages and cultures [14,15,16].

Social capital may explain some of the health disparities that exist among social groups, such as self-rated health, psychological health, and psychological distress [17]. High social capital may reduce barriers to a healthy lifestyle, increase awareness to physical activity (PA) and healthy eating habits, and provide the resources needed to engage in PA regularly [18,19]. A recent study in Israel focused on Jewish women participating in a cachibol league (in which a sport similar to volleyball is played), the study included the participants and a comparison group and followed the women for more than one year after joining the team. Participants in the league had higher levels of social capital and well-being compared to the comparison group. In addition, the authors found an increase in two social capital measures-social support and social involvement during the follow up period, compared to no change in the comparison group [20]. Therefore, this study concluded that women who participated in the league may have had higher social capital to begin with, and that during the year of participation in the cachibol league, certain aspects of their social capital improved [20].

The development of social capital is especially important in poor, deprived, or segregated communities because of the lack of resources and social networks within the community [5]. In Israel, there are inequalities in social capital and health between the country’s two major population groups: Jews and Arabs. There are 9.2 million Jews and Arabs residing in Israel; the Arab population comprises 21% of the population [21]. Previous literature has shown that social capital is higher among Jews as compared to Arabs: Jews reported higher levels of social trust, perceived helpfulness, trust in authorities, and social support compared to Arabs [22,23]. These inequalities could be the result of socioeconomic and cultural differences between the two ethnic groups as well as institutional discrimination of Arabs [23,24]. As a whole, Arabs have lower levels of education and income compared to the Jewish population, and Arab neighborhoods are characterized by higher rates of poverty, crime, and violence. Furthermore, since the establishment of the state of Israel, there has been a segregation of services and discrimination in allocating resources to the Arab population, which has resulted in a lack of opportunities and lower levels of investment made in the educational and health care among the Arab population [23,24,25].

In addition, health indicators in Israel present a picture of poorer health in the Arab population compared to the Jewish population [23]. Although it is improving, there are still higher rates of chronic illnesses and a lower life expectancy in the Arab population compared to the Jewish population [26].

One health risk factor that may add to the disparities in health between Jews and Arabs could be PA, where disparities have been reported between Jewish and Arab women. According to the Israeli Ministry of Health, Arab women report a lower rate of regular leisure PA (42%) compared to Jewish women (59%). In addition, only 24% of Arab women reported exercising for at least 30 consecutive minutes three times a week or more compared to 33% among Jewish women [27].

There are many barriers for conducting PA among women, including time and resource limitations and necessary childcare [28,29]. For women from lower socioeconomic status (SES) and traditional cultures, these barriers are amplified by the lack of access to PA facilities, fewer resources, more traditional gender roles, and cultural expectations [1,30,31].

Team sports include activities that require teamwork, socialization, and commitment to PA, all of which are associated with social capital [32]. Participation in team sports among women can provide a sense of security; support; and promote positive social interactions, intimacy and social inclusion, more so compared to individual sports such as running or going to the gym [16,33,34,35].

Participation in team sports can also contribute to better physical and mental health [36]. It reduces stress and increases the intrinsic motivation and persistence in PA, due to the enjoyment and social interactions it involves [35,37,38]. Previous studies have shown that women who participate in group sports report decreased physical symptoms such as headaches, abdominal pain, and back pain [35].

Team sports have previously been used as a method for creating positive associations between communities and bridging differences between cultures and ethnicities [39]. As women do not have the resources, motivation, or encouragement from their surroundings in order to engage in PA, group sports can provide them with the social structure that is needed in order to improve their health and well-being and eliminate some PA barriers [40].

Even though there is evidence that team sports may increase social capital, which in turn contributes to an active and healthy lifestyle, there is a paucity of data regarding the advantages of team sports on the health and well-being of women from diverse backgrounds. Baron-Epel et al. [20] found increased social support and social involvement among women who participated in a team sports league when compared to those who did not participate, however their study included only Jewish women of relatively higher socioeconomic status and did not take into consideration women from diverse backgrounds.

### The Israeli Mamanet Cachibol League

Cachibol (also known as Newcomb Ball) is a team ball game that has characteristics similar to volleyball. The game was invented in 1895 by Clara Gregory Baer, a physical education pioneer at Sophie Newcomb College in Louisiana, USA [41]. In both cachibol and volleyball, the aim is to get the ball into the opposing court by transferring it across the net. However, in cachibol the players are allowed to catch and hold the ball for up to one second instead of volleying, hitting or striking it. The characteristics of the game allows players of any age or level of fitness to participate in the sport; thus, cachibol is becoming increasingly popular, particularly among middle-aged women [42].

The Mamanet Cachibol League (MCL) was established in 2005 as a community model that incorporates PA, sports education, social networks, and support, among women from different population groups. In Israel, MCL currently encompasses thousands of players in over 90 Arab and Jewish municipalities throughout the country. The MCL teams have a weekly practice and they compete against each other in local and national tournaments. Participants in MCL attend local and national community events, including activities that involve spouses and children of the participants. MCL is also active internationally in various countries including Canada, Cyprus, Finland, Germany, Greece, Italy, Singapore, and the United States (https://www.mamanet.org.il/International.asp).

The purposes of the present study were:

1. To assess whether participants in MCL had higher social capital and well-being compared to non-participants (comparison group), among both Arab women and Jewish women.
2. To assess whether social capital was associated with well-being (SRH, psychosomatic and depressive symptoms) among both Jewish and Arab women.

## 2. Materials and Methods

We performed a quantitative cross-sectional study among a sample of 381 MCL participants from the Arab and Jewish sectors: 198 MCL participants (102 Jews and 96 Arabs) and 183 non-participants (comparison group; 102 Jews and 81 Arabs). An online questionnaire was delivered to participants and included information about health indicators (self-reported health), psychological characteristics (psychosomatic and depressive symptoms), and social capital (trust, social support, and social involvement). Arab and Jewish women who did not participate in MCL but had similar sociodemographic characteristics to the MCL participants were used as a comparison group.

### 2.1. Sampling Method

The sample of Jewish participants was recruited with the help of the MCL organization, which provided names and phone numbers of the teams’ captains, to whom the online questionnaire was sent. The captains sent out the online questionnaire via the WhatsApp application (text message) to all of their team’s members.

The sample of Arab participants was also recruited with the help of the MCL organization, but unlike the Jewish MCL participants, the Arab participants filled out a “pen and paper” questionnaire delivered by a research assistant. The reason the Arab participants filled out a printed version of the questionnaire was due to the fact that many did not own smart phones and were therefore unable to access the questionnaire online via their cell phone. Previous research has suggested only minor differences between online and paper questionnaires [43,44,45].

The sampling method was stratified sampling. MCL management provided the list of active teams that have been active for at least one year across Israel. A total of 10 teams were sampled from the Jewish sector, which included 102 participants. Ten teams from the Arab sector were sampled, which included 96 participants. Teams from different geographic regions and socio-economic levels (low, medium, high) according to the Israeli Central Bureau of Statistics [46] were included. For the comparison group, a sample of 177 Jewish women and 81 Arab women was randomly sampled from an Internet panel that included 100,000 people. The random sampling was intended to result in a comparison group that would have the same demographic and socio-economic and geographic characteristics as those of the MCL participants. sample and they did not participate in any organized team sport.

### 2.2. Instruments and Measures

The questionnaire was built for the study purposes and was based on valid questionnaires from the scientific literature [47,48,49,50]. The questionnaire was delivered either online or in printed form and included information on social capital, self-reported health, body mass index (BMI), and psychosomatic and depressive symptoms. A principal component factor analysis with varimax rotation was calculated for the social capital index, and internal consistencies (Cronbach α) were calculated for the study variables.

#### 2.2.1. Dependent Variables

1. Self-reported health (SRH) [48]: Self-reported health was measured using the standard question: “How do you evaluate your health generally?” Answers range between 1 and 6 with 1 = very poor and 6 = excellent. Self-reported health was exponentially transformed due to a non-normal distribution (skewness = −0.97, SE = 0.12).
2. Psychosomatic symptoms [49]: Psychosomatic symptoms were evaluated with a six-item checklist, including symptoms experienced in the past six months: stomachache, headache, backache, irritability or bad temper, nervous, and dizziness. Answers ranged between 1 and 5 with 1 = rarely or never and 5 = about every day. An overall psychosomatic symptom index was calculated from the means of the items. Internal consistency was α = 0.78, with higher scores representing a greater extent of experienced symptoms. Psychosomatic symptoms were log transformed due to non-normal distributions.
3. Depressive symptoms [50]: The scale for depressive symptoms was based on the Personal and Social Development Survey. It included eight items that were potentially experienced in the past week (e.g., depressed, lonely, sad, inability to do things). Answers ranged between 1 = not at all or almost not at all and 4 = all the time or almost all the time. An overall depressive symptom index was calculated from the mean of the items. Internal consistency was α = 0.82, with higher scores representing a greater extent of experienced depressive symptoms. Depressive symptoms were log transformed due to non-normal distributions.

#### 2.2.2. Independent Variables

1. Participation in MCL was measured by participation or non-participation (yes/no).
2. Demographic characteristics: Demographic characteristics included age, number of children, family status (married/in a relationship), education level (academic, non-academic education), and family income (below average, average, above average).
3. Body mass index: BMI was calculated according to participants’ reports about weight and height and was used as a control variable for self-reported health.
4. Social capital [47]: Social capital comprised of three sub-scales (trust, social support, and social involvement) that were yielded through a process of principal component factor analysis with varimax rotation. Answers ranged between 1 and 5, with higher scores representing greater social capital.

The first component, social trust, was comprised of 3 items including: “In general, do you think that most people can be trusted, or that you can’t be too careful in dealing with people?” and “In your opinion, would most people try to take advantage of you if given a chance, or would they try to be fair to you?” Eigenvalue was 3.67, explaining 30.59% of the variance. Loadings ranged between 0.77 and 0.85, and internal consistency was α = 0.82.

The second component, social support, was comprised of 5 items including: “’How many close friends do you have?” and “To what extent do you feel appreciated by society?” Eigenvalue was 1.79, explaining 14.89% of the variance. Loadings ranged between 0.54 and 0.72, and internal consistency was α = 0.69.

The third component, social involvement, was comprised of 4 items including: “To what extent have you participated in any community event in the past six months?” and “To what extent are you likely to meet friends or acquaintances when you go out shopping in your area of residence?” Eigenvalue was 1.19, explaining 9.89% of the variance. Loadings ranged between 0.47 and 0.76, and internal consistency was α = 0.60. The generally agreed upon lower limit for Cronbach’s alpha is 0.70. However, in exploratory research it may decrease to 0.60 [51]. Confirmatory factor analysis has verified the factor structure of the scale: CFI = 0.942, RMSEA = 0.058, NFI = 0.904, NNFI = 0.904. Inter-correlations between the three sub-scales ranged between r = 0.17 and r = 0.49 (*p* < 0.001), and thus a total score for social capital was composed as well (α = 0.78).

### 2.3. Data Analysis

Background characteristics were described by group and ethnicity, with frequencies and percent, means and standard deviations, and compared with Z tests and analyses of variance. Skewed variables were log or exponentially transformed. The mean scores of the main study variables are presented (Table 2); the associations between them were examined using the Pearson correlation (Table 2). Analyses of covariance were calculated for the study variables, by group and ethnicity, controlling for age, education level, and BMI (Table 3). Significant interactions between group and ethnicity were interpreted with estimated marginal means. Multiple regressions with sequential entry of the variables were calculated for SRH, psychosomatic symptoms, and depressive symptoms (Table 4). The first step included group (1-MCL, 0-control), ethnicity (1-Jewish, 0-Arab), age, and education level (1-academic, 0-non-academic); BMI was included regarding SRH only. Step 2 included the dimensions of social capital. All data were analyzed in SPSS version 26 (IBM SPSS Statistics for Windows, 2019) [52].

## 3. Results

The sample included a total of 381 participants: 198 MCL participants (102 Jews and 96 Arabs) and 183 women in the comparison group (102 Jews and 81 Arabs; Table 1). Participants were 25 to 64 years old (mean: 39.78; standard deviation [SD]: 6.27), with Jewish women in the MCL being the oldest and Arab women in the comparison group being the youngest. The mean number of children was 2.79 (SD = 1.03), with no group or ethnic differences. The average BMI was 26.24 (SD = 4.82), with a significant ethnic difference, such that Jewish participants had lower BMIs compared to Arab participants. Just over 90% of the participants were married or in a relationship, with no group or ethnic differences. Academic education was more prevalent among Jewish compared to Arab participants, and above average family income was more prevalent among Jewish compared to Arab participants.

Table 2 describes the mean scores and associations between the dependent variables among the MCL participants by ethnicity. The overall score of social capital and its sub-scales were moderate, except for social support, which was higher among both Jewish and Arab MCL participants. SRH was high, and psychosomatic and depressive symptoms were in the low to below range. Negative correlations were found between social capital and depressive symptoms among both Jewish and Arab MCL participants. SRH was negatively associated with psychosomatic and depressive symptoms among Jewish MCL participants, and the latter two were positively correlated in both groups.

Correlations between the study variables and the women’s ages were significant and positive for social capital (r = 12; *p* = 0.016 to r = 0.23; *p* < 0.001), and significant and negative for psychosomatic and depressive symptoms (r = −0.18 to r = −0.24; *p* < 0.001). They were positive for academic education with social capital (r = 0.12; *p* = 0.018 to r = 0.19; *p* < 0.001), and negative for depressive symptoms (r = −0.22; *p* < 0.001). Family income was highly correlated with education level (r = 0.46 *p* < 0.001). Lastly, BMI was negatively associated with SRH (r = −0.14 *p* = 0.008). Thus, the study hypotheses were examined while controlling for age and education level (0-non-academic, 1-academic). Analyses regarding SRH were controlled for BMI as well. Analyses of covariance were calculated to assess differences in the study variables by group and ethnicity, controlling for age and education level.

Results in Table 3 reveal significant group differences in all variables. MCL participants had higher social capital than the comparison group in terms of trust, social support, social involvement, and the total score. We found that participation in MCL was most strongly related to the items that included social involvement (each item on its own and also when grouped together) as compared to social support and trust. They reported better well-being in terms of higher SRH, lower psychosomatic, and lower depressive symptoms, compared to the comparison group. Ethnic differences were found for the social capital sub-scales of trust and social support, showing higher levels of these dimensions among Jewish participants than Arab participants. Depressive symptoms were higher among Arab than Jewish participants.

A significant interaction between group and ethnicity was found in depressive symptoms. Among the Jewish participants, the MCL group had lower depressive symptoms compared to the comparison group (F (1, 375) = 26.52; *p* < 0.001; η2 = 0.069). However, there was no significant difference between MCL and the comparison group among the Arab participants (F (1, 375) = 1.98; *p* = 0.160; η2 = 0.005).

Multiple linear regressions with sequential entry of the variables were calculated to assess the extent to which MCL participation, ethnicity, the background variables, and social capital were associated with SRH, psychosomatic symptoms, and depressive symptoms, as shown in Table 4.

Results revealed that all regression models were significant. The explained variance ranged between 8% (SRH) to 32% (depressive symptoms). Group was significant for all three regressions. That is, SRH was higher for MCL participants than non-participants, and psychosomatic and depressive symptoms were lower. Ethnicity was significant for SRH and depressive symptoms. That is, both SRH and depressive symptoms were higher for Arab women than Jewish women.

Beyond the background variables, better SRH was associated with lower BMI and higher social support. Experiencing fewer psychosomatic and depressive symptoms was associated with higher trust and social support, beyond group and ethnicity. Lower depressive symptoms were further associated with higher social involvement.

## 4. Discussion

Our findings suggest that Jewish women (including MCL participants) have higher levels of social capital (trust and social support) compared to Arab women (including MCL participants). These differences in social capital between the two ethnic groups are consistent with previous literature that point to the effects of socioeconomic and cultural differences, such as lower education and income levels and higher poverty, crime and violence levels among the Arab population in Israel [22,23,24]. In addition, we found that Jewish women (including MCL participants) have lower depressive symptoms compared to Arab women. These differences between the two ethnic groups are also consistent with previous literature [23,53] suggesting they are a result of Arab culture, social factors and institutional discrimination [54].

Concurrently, the findings also suggest that both Jewish and Arabs participants in MCL had higher levels of social capital (trust, social support, and social involvement) and well-being (higher SRH, and lower levels of psychosomatic and depressive symptoms) compared to each of the comparison groups. Previous literature suggests that social connections, social interactions, and social networks involving amateur team sports contribute to the development of social capital among participants [20,29,55,56]. An amateur team sports league serves as a platform for social activity, which includes traveling together as a team, participating in major sporting events, and partaking in recreational events together, all of which contribute to group coherence [28].

Findings related to higher levels of social capital among MCL participants relative to the comparison groups may also be related to MCL participants being more inclined to participate in social activities compared to non-participants. In a pre-post study design of Jewish women, Baron-Epel et.al found that social support and social involvement were higher to begin with among women who participated in an MCL program when compared to women who did not, therefore it is reasonable that these findings are similar [20]. Accordingly, in reviewing the different measures of social capital we found that social involvement was most strongly related to participation in MCL as compared to social support and trust, pointing to possible higher levels of social engagement.

The disparities in well-being between MCL participants and non-participants may suggest that that MCL participants feel physically and mentally better than non-participants. Previous studies have suggested a positive connection between participation in PA and health status [57,58,59]. Physical inactivity, on the other hand, can negatively affect one’s mood, as well as cause aches and pains [60]. Furthermore, low levels of psychosomatic symptoms (e.g., abdominal pain or headache), low depressive symptoms, and high general well-being have previously been identified among women who participated in PA [35,57,58,61,62]. Baron-Epel et al. [20] found that women joining MCL reported higher well-being (better SRH and lower psychosomatic and depressive symptoms) compared to women not joining MCL. It might suggest that women who are physically and mentally healthier may be more inclined to take part in this type of sport because of its physical and cooperative nature.

An interaction between group (MCL/non-MCL participants) and ethnicity (Arab/Jewish women) showed that Jewish participants in MCL had lower depressive symptoms compared to the comparison group, but there was no such significant difference among Arab participants in MCL compared to the comparison group. The Israeli National Health Interview Survey 2003–2004, found that the rate of depression among Arab women was 1.7 times higher than that of Jewish women [53]. Kaplan et al. [63] found that the level of depression among Arab women was higher than Jewish women and rose with age. Arab women depression rates rose steadily from 14% in the age group of 26-35 to 51.7% at the age group of 56+, while among Jewish women the rate rose from 7.7% in the youngest group to 15.1% among the age group of 56+.

A number of factors may be related to the lack of improvement in depressive symptoms despite participation in MCL. Various cultural and social factors have been found to be related to mental health and well-being of Arab women, including community values [54], rigid hierarchical structure [64], gender (male over female) and age (older over younger) inequalities [65]. Arab society also endorses collectivism, including a significant concern for maintaining traditional associations [65,66,67]. Individuals are interdependent, mainly within the family group, and are expected to behave in the manner that is anticipated within their respective group [54]. Despite the changes in the Arab community, with women becoming stronger in terms of employment outside the home and financial independence, the patriarchal power structure is still common [67]. Women are expected to serve as caregivers, play active roles in the household, and look after family members. Research has found that women are likely to lose the conditional support of their family if they do not meet the above expectations [54,63].

These sociocultural norms and values can be significant barriers to Arab women’s participation in sports and PA. Specifically, several studies point to impediments that limit participation in sports and PA for Arab women in Qatar [68,69], Malaysia [70], and Turkey [71]. These include cultural beliefs, dress code requirements (for Arab Muslims), risk of reputation, gender segregation, family prohibitions, women’s roles, and family responsibilities. Koca et al. [71] emphasized that lack of time caused by family responsibilities as a major constraint to Arab women’s participation in recreational PA. However, the effect of the Arab sociocultural norms and values on women who do participate in PA and sports and the price that they pay in terms of stress and other mental health issues is yet unclear and require further research.

Another purpose of this study was to assess the associations between social capital and well-being among Jewish and Arab participants in MCL. Our findings suggest that associations exist between social capital and well-being, beyond ethnicity and group. These findings are consistent with previous literature suggesting that social connections are a leading source of human well-being [72,73]. More specifically, social support was found to be associated with lower levels of depressive symptoms [74], as well as with the reduction of psychological distress such as depression or anxiety during times of stress [75]. Social trust at the individual level is linked to reduced levels of depressive symptoms and improved psychological well-being [76,77,78]. Social involvement was also found to be connected to well-being. Similarly, people who volunteer more frequently are both healthier and happier than those who do not volunteer [79,80].

Several limitations of the study should be noted. Firstly, there is an issue with causality regarding social capital and well-being, as the cross-sectional design of the study involved no possibility to distinguish the directions of cause and effect. Women who participated in MCL might have tended to be more socially oriented than non-participants. Secondly, the distribution of MCL in the Arab society in Israel is relatively low. Less than 5% of MCL participants are Arab, when Arabs are nearly 21% of the general Israeli population. The sample of the Arab MCL participants was in fact a small convenience sample, chosen from a small number of teams. Finally, this was not an intervention study with a random assignment of women to comparison and intervention groups. Therefore, it would be logical to claim that the women who participated in MCL may have been more socially inclined to join, and possibly had higher levels of social capital to begin with.

## 5. Conclusions

In conclusion, our findings suggest that amateur team sports such as MCL are related with higher levels of well-being and social capital, in particular social involvement. Future research should focus on longitudinal studies that examine the change in social capital and well-being over time, particularly among Arab participants.

## Figures and Tables

**Table 1:** Background characteristics according to ethnicity and group (*n* = 381).

		Total *M* (*SD*)	Jews		Arabs		*F*_group_ (1, 375) (*p*) (η^2^)	*F*_ethnic_ (1, 375) (*p*) (η^2^)	*F*_group × ethnic_ (1, 375) (*p*) (η^2^)
			MCL *M* (*SD*) (*n* = 102)	Comp.*M* (*SD*) (*n* = 102)	MCL *M* (*SD*) (*n* = 96)	Comp. *M* (*SD*) (*n* = 81)			
**Age, in Years**	25−64	39.78 (6.27)	42.73 (5.95)	40.30 (4.73)	40.07 (5.95)	35.11 (4.99)	38.74 (*p* < 0.001) (η^2^ = 0.095)	43.81 (*p* < 0.001) (η^2^ = 0.107)	4.58 (*p* = 0.033) (η^2^ = 0.012)
**Number of Children**	1−7	2.79 (1.03)	2.76 (0.79)	2.97 (1.01)	2.80 (1.37)	2.60 (0.79)	0.01 (*p* = 0.964) (η^2^ = 0.001)	2.38 (*p* = 0.124) (η^2^ = 0.006)	3.63 (*p* = 0.057) (η^2^ = 0.010)
**BMI**	17.6−41.6	26.24 (4.82)	25.71 (4.64)	25.01 (4.44)	27.72 (4.31)	26.77 (5.57)	2.81 (*p* = 0.094) (η^2^ = 0.008)	14.81 (*p* < 0.001) (η^2^ = 0.038)	0.06 (*p* = 0.800) (η^2^ = 0.001)
		***N* (%)**	***N* (%)**	***N* (%)**	***N* (%)**	***N* (%)**			
**Family Status**	**Married/ in a relationship**	342 (90.7)	90 (88.2)	93 (91.2)	86 (93.5)	73 (90.1)	*Z* = 0.01 (*p* = 0.997)	*Z* = 0.73 (*p* = 0.463)	--
**Education**	**Academic**	237 (63.0)	75 (73.5)	85 (83.3)	31 (34.1)	46 (56.8)	*Z* = 3.34 (*p* = 0.001)	*Z* = 6.74 (*p* < 0.001)	--
**Family Income**	**Above Average**	153 (41.9)	53 (52.0)	69 (67.6)	17 (21.3)	14 (17.3)	*Z* = 1.33 (*p* = 0.182)	*Z* = 7.79 (*p* < 0.001)	--

Abbreviations: M, mean; SD, standard deviation; MCL, Mamanet Cachibol League; Comp., Comparison group BMI, body mass index.

**Table 2:** Mean scores and correlations between the study variables among MCL participants, by ethnicity.

	**Jewish MCL (*n* = 102)**						
	***M*** **(*SD*)**	**2.**	**3.**	**4.**	**5.**	**6.**	**7.**
1. Trust (1−5)	3.63 (0.67)	0.48 ***	0.07	0.71 ***	−0.04	−0.01	−0.23 *
2. Social support (1−5)	4.29 (0.56)	-	0.16	0.72 ***	0.03	−0.13	−0.20 *
3. Social involvement (1−5)	3.25 (0.79)		-	0.66 ***	0.03	0.13	−0.12
4. Total social capital (1−5)	3.72 (0.47)			-	.01	0.02	−0.26 **
5. Self-reported health (SRH) (1−6)	5.15 (0.74)				-	−0.37 ***	−0.28 **
6. Psychosomatic symptoms (1−5)	1.90 (0.56)					-	0.22 *
7. Depressive symptoms (1−4)	1.51 (0.28)						-
	**Arab MCL (*n* = 96)**						
	***M*** **(*SD*)**	**2.**	**3.**	**4.**	**5.**	**6.**	**7.**
1. Trust (1−5)	2.65 (0.81)	0.18	0.12	0.65 ***	−0.13	0.02	−0.16
2. Social support (1−5)	3.84 (0.71)	-	0.34 ***	0.70 ***	0.06	−0.23 *	−0.27 **
3. Social involvement (1−5)	3.42 (0.80)		-	0.72 ***	−0.03	−0.16	−0.24 *
4. Total social capital (1−5)	3.32 (0.55)			-	−0.03	−0.15	−0.32 **
5. Self-reported health (SRH) (1−6)	5.16 (0.91)				-	−0.13	−0.16
6. Psychosomatic symptoms (1−5)	2.02 (0.58)					-	0.36 ***
7. Depressive symptoms (1−4)	2.04 (0.51)						-

Note: * *p* < 0.05; ** *p* < 0.01; *** *p* < 0.001. Abbreviations: MCL, Mamanet Cachibol League; M, mean; SD, standard deviation.

**Table 3:** Means, standard deviations, and F values for group and ethnic differences in the study variables (n = 381).

	Jewish Women (*n* = 204)			Arab Women (*n* = 177)			MCL *M* (*SD*) (*n* = 198)	Comp*.M* (*SD*) (*n* = 183)	*F*_group_ (1, 375) (*p*) (η^2^)	*F*_ethnic_ (1, 375) (*p*) (η^2^)	*F*_group × ethnic_ (1, 375) (*p*) (η^2^)
	MCL *M* (*SD*) (*n* = 102)	Comp. *M* (*SD*) (*n* = 102)	Total *M* (*SD*)	MCL *M* (*SD*) (*n* = 96)	Comp*. M* (*SD*) (*n* = 81)	Total *M* (*SD*)	Total *M* (*SD*)	Total *M* (*SD*)			
Trust	3.63 (0.67)	3.29 (0.90)	3.46 (0.81)	2.65 (0.81)	2.64 (0.87)	2.65 (0.83)	3.17 (0.89)	3.00 (0.94)	4.23 (*p* = 0.041) (η^2^ = 0.012)	58.83 (*p* < 0.001) (η^2^ = 0.141)	3.31 (*p* = 0.070) (η^2^ = 0.009)
Social Support	4.29 (0.56)	3.94 (0.64)	4.12 (0.62)	3.84 (0.71)	3.55 (0.74)	3.70 (0.74)	4.07 (0.68)	3.77 (0.71)	23.48 (*p* < 0.001) (η^2^ = 0.061)	16.47 (*p* < 0.001) (η^2^ = 0.044)	0.01 (*p* = 0.950) (η^2^ = 0.001)
Social Involvement	3.25 (0.79)	2.74 (0.96)	2.99 (0.91)	3.42 (0.80)	2.78 (0.76)	3.12 (0.84)	3.33 (0.80)	2.75 (0.88)	36.09 (*p* < 0.001) (η^2^ = 0.091)	2.12 (*p* = 0.146) (η^2^ = 0.006)	0.44 (*p* = 0.507) (η^2^ = 0.001)
Total Social Capital	3.72 (0.47)	3.32 (0.64)	3.52 (0.60)	3.32 (0.55)	2.99 (0.61)	3.17 (0.60)	3.53 (0.55)	3.17 (0.65)	36.61 (*p* < 0.001) (η^2^ = 0.092)	20.03 (*p* < 0.001) (η^2^ = 0.053)	0.14 (*p* = 0.710) (η^2^ = 0.001)
Self-Reported Health (SRH)	5.15 (0.74)	4.69 (0.95)	4.92 (0.88)	5.16 (0.91)	4.86 (1.16)	5.02 (1.04)	5.15 (0.82)	4.77 (1.05)	17.39 (*p* < 0.001) (η^2^ = 0.047)	2.37 (*p* = 0.125) (η^2^ = 0.007)	0.05 (*p* = 0.818) (η^2^ = 0.001)
Psychosomatic Symptoms	1.90 (0.56)	2.32 (0.70)	2.11 (0.66)	2.02 (0.58)	2.70 (0.90)	2.34 (0.82)	1.96 (0.57)	2.49 (0.82)	42.44 (*p* < 0.001) (η^2^ = 0.106)	2.67 (*p* = 0.103) (η^2^ = 0.007)	1.48 (*p* = 0.225) (η^2^ = 0.004)
Depressive Symptoms	1.51 (0.28)	1.80 (0.44)	1.65 (0.39)	2.04 (0.51)	2.10 (0.59)	2.07 (0.55)	1.76 (0.49)	1.93 (0.53)	18.64 (*p* < 0.001) (η^2^ = 0.049)	41.89 (*p* < 0.001) (η^2^ = 0.104)	5.57 (*p* = 0.019) (η^2^ = 0.015)

Note: Abbreviations: MCL, Mamanet Cachibol League; M, mean; SD, standard deviation; Comp., Comparison group.

**Table 4:** Multiple regression coefficients (β) for self-reported health, psychosomatic symptoms, and depressive symptoms, with background variables and social capital (n = 381).

	Self-Reported Health (SRH)	Psychosomatic Symptoms	Depressive Symptoms
Step 1			
Group	0.22 ***	−0.33 ***	−0.23 ***
Ethnicity	−0.12 *	−0.09	−0.34 ***
Age	−0.13 *	−0.11 *	−0.03
Education level	0.02	−0.06	−0.14 **
BMI	−0.19 ***	--	--
*Adj.R* ^2^	0.078 ***	0.148 ***	0.209 ***
Step 2			
Group	0.20 ***	−0.27 ***	−0.12 *
Ethnicity	−0.14 *	−0.02	−0.22 ***
Age	−0.14 *	−0.10	−0.01
Education level	0.01	−0.04	−0.10 *
BMI ^1^	−0.19 ***	--	--
Trust	−0.03	−0.12 *	−0.19 ***
Social support	0.15 *	−0.13 *	−0.20 ***
Social involvement	−0.04	−0.05	−0.11 *
*Adj.R* ^2^	0.085 ***	0.182 ***	0.320 ***
*F* (7, 373)	5.14 ***	12.51 ***	25.39 ***

Note: * *p* < 0.05; ** *p* < 0.01; *** *p* < 0.001. ^1^ BMI was used as a control variable in relation with Self-Reported Health only. Abbreviations: BMI; body mass index.

## Data Availability

The data that support the findings of this study are available from the corresponding author, upon reasonable request.
